# The effect of endometrial thickness on pregnancy outcomes of frozen-thawed embryo transfer cycles which underwent hormone replacement therapy

**DOI:** 10.1371/journal.pone.0239120

**Published:** 2020-09-24

**Authors:** Zhang Shaodi, Li Qiuyuan, Yin Yisha, Zhang Cuilian

**Affiliations:** Reproductive Medical Center, Henan Provincial People’s Hospital (People’s Hospital of Henan University, People's Hospital of Zhengzhou University), Zhengzhou, Henan Province, People’s Republic of China; Peking University Third Hospital, CHINA

## Abstract

**Objective:**

To investigate the impact of endometrial thickness on the embryo transfer(ET) day on the clinical pregnancy outcomes of frozen-thawed embryo transfer cycles which have undergone hormone replacement therapy(HRT-FET).

**Methods:**

A total of 10,165 HRT-FET cycles performed between January 2013 to December 2017 in the Reproductive Medicine Center of Henan Provincial People’s Hospital were studied retrospectively. All patients were grouped according to their endometrial thickness on the ET day (each group having an increment of 1mm between two neighboring groups). Multivariate regression analysis, curve fitting and threshold effect analysis were performed on all data.

**Results:**

After adjusting for the age, duration of infertility, body mass index(BMI), infertility type and number and type of embryos transferred, a significant correlation was observed to be between the endometrial thickness and implantation rates (aOR: 1.08; 95% CI: 1.06–1.10, p < 0.0001), clinical pregnancy rate(aOR: 1.10; 95% CI: 1.07–1.14, p < 0.0001)and live birth rate (aOR: 1.09; 95% CI: 1.06–1.12, p < 0.0001). The numerical value of the cut-off point for the endometrial thickness was 8.7 mm. When the endometrial thickness was less than 8.7 mm, with each additional 1 mm of endometrial thickness, the implantation rate increased by 32%, the clinical pregnancy rate increased by 36%, and the live birth rate increased by 45%.

**Conclusions:**

In the HRT-FET cycles, the optimal live birth rate would be obtained when the endometrial thickness remains within the range of 8.7–14.5 mm. If the endometrium is too thin or too thick, the live birth rate will be reduced.

## Introduction

Embryo implantation requires a cross talk between the embryo and the receptive endometrium. The synchrony between embryo development and endometrium is the key to success in assisted reproductive technology (ART) [1]. In addition to embryo quality, endometrial receptivity is widely accepted as one of the main limiting factors for ART outcomes, especially in frozen–thawed embryo transfer (FET) cycles.

For FET, hormone replacement therapy (HRT) exogenous estrogen and progesterone (P) support has been widely used in recent years in clinical practice for endometrial preparation because of its benefit to women with irregular menstrual cycles. With menstrual regularity the date of FET can be scheduled in advance compared to using a natural cycle [2, 3]. There are many factors such as an LH surge, HCG administration, and serum P levels that affect the endometrial implantation window during the natural cycle-FET (NC-FET) cycles. But in HRT-FET cycles, the major factor affecting the endometrial implantation window is the time of progesterone administration.

Endometrial thickness (EMT) is the most widely used prognostic indicator for measuring endometrial receptivity. Although observations suggest that the association between EMT and IVF outcomes is still contradictory and inconsistent [4, 5], there is a general consensus that thin endometrium is associated with low pregnancy rate. Furthermore, several studies have shown that there is a correlation between EMT and clinical pregnancy outcome after conducting FET [6, 7]. However, very little research has focused on the relationship between endometrial thickness and clinical outcomes of the HRT-FET cycle.

Therefore, in order to understand what the optimal endometrial thickness for implantation rate and clinical pregnancy rate or live birth rate after undergoing HRT-FET and to evaluate the effect which endometrial thickness has on subsequent outcomes, we retrospectively analyzed the data of 10,165 HRT-FET cycles in the Reproductive Medicine Center of Henan Provincial People’s Hospital from January 2013 to December 2017.

## Materials and methods

### Subjects

The study was reviewed and approved by the Ethics Committee of People’s Hospital of Zhengzhou University. This study is a retrospective cohort study, consequently, it is exempt from the informed consenting process. In this study, patient’s medical records were anonymized and de-identified prior to the time data was collected from our electronic database, Clinical Reproductive Medicine Management System and the Wuhan Huchuang Reproductive Center Electronic Medical Record Management System.

During the period between January 2013 and December 2017, a total of 10,165 HRT-FET cycles in the Reproductive Medicine Center of Henan Provincial People’s Hospital were studied retrospectively. Inclusion criteria were set as follows. (1) The endometrium is prepared using HRT protocol; (2) at least one good quality embryo was transferred during each cycle. Exclusion criteria on the other hand, included the followings: (1) Chromosomal abnormality in either partner; (2) uterine malformation; (3) intrauterine conditions affecting the pregnancy outcomes of FET, such as endometrial polyps, uterine cavity adhesion, history of endometrial tuberculosis, hydrosalpinx with a reflux into uterine cavity; (4) cycles which underwent pre-implantation genetic testing (PGT); and (5) cycles of spontaneous ovulation. All patients were grouped according to the endometrial thickness on the embryo transfer day with a 1 mm interval between each group. Multivariate regression analysis, curve fitting and threshold effect analysis were performed on all cycles.

### Endometrial preparation protocol

HRT-FET cycles: Patients started oral estradiol with a total daily dosage of 4–8 mg (Progynova, Bayer, Leverkusen, Germany), taken twice a day on day 2–3 of the cycle. This dose was adjusted every 4 days according to the endometrial thickness. Progesterone would be administered when the endometrial thickness reached 8 mm, approximately on day 12–20 of the cycle. For patients who had a thin endometrial thickness during their past cycles, we would increase the dose and duration of estrogen application. If the endometrial thickness reached the same level it had been on the HCG trigger day in a controlled ovarian stimulation cycle, progesterone will be given to transform endometrium. The luteal phase support (LPS) included a daily dose of 90 mg of vaginal progesterone gel, or a dose of 200 mg of micronized natural progesterone (Utrogestan, LaboratoiresBesins International SA, France) three times a day. In addition to this, a dose of 20 mg of oral dydrogesterone was given once a day. LPS continued for at least 8 weeks or till the hCG test result turned out negative. Embryos were transferred on the fourth day (cleavage stage) or on the sixth day (blastocyst) after progesterone was administered.

### Measuring the endometrial thickness

Trans-vaginal ultrasound scanning (SSD-ALPHA7, ALOKA, HITACHI, Japan) with a 2D, 6.67MHz probe was performed on the ET day. The Measurements of the endometrial thickness of each patient were conducted according to SOP before embryos were transferred by two experienced sonographers using transvaginal ultrasound. Measurements were carried out, they were noted in millimeters (mm).

### Embryo transfer and embryo score

Prior to ET, each embryo were was graded according to their developmental speed, degree of fragmentation and the evenness of cleavage sphere. The embryos with a 7 ~ 9 blastomere, uniform cytoplasm, regular morphology, fragmentation < 10% were considered to be high-quality embryos. Blastocyst scoring was performed by following the Gardner scoring system [8], and embryos graded 3BB or better were defined as good quality embryos.

In order to assess FET outcomes, serum human chorionic gonadotropin (hCG) was measured 14 days after an embryo transfer was conducted. The dose of estrogen and progesterone remained the same till 14 days after embryo transfer, and then was gradually reduced after the fetal heart examination, and completely stopped at gestational week 10. If the serum hCG was positive, an ultrasound examination was performed 2–3 weeks later to confirm intrauterine pregnancy and determine the number of gestational sacs. The diagnosis of ectopic pregnancy was made by observing the extra-uterine gestational sac during ultrasonography or laparotomy/laparoscopic surgery. A spontaneous miscarriage was defined as a spontaneous pregnancy loss after sonographic visualization of an intrauterine gestational sac. A clinical pregnancy was defined by at least one gestational sac found on the ultrasonography 4–6 weeks after an embryo transfer. An ectopic pregnancy and spontaneous miscarriage were all considered as clinical pregnancies. A miscarriage that occurred before gestational Week 12 was defined as an early miscarriage. A live birth was defined as one or more live babies delivered after 28 weeks of gestation.

### FET cycle outcomes

The primary outcome included the live birth rate, gestational week at the delivery and newborn birth weight. The secondary cycle outcomes included the implantation rate, clinical pregnancy rate, early miscarriage rate, and ectopic pregnancy rate. Implantation rate = number of clinical gestational sacs / total number of embryos transferred *100%. Clinical pregnancy rate = number of cycles with clinical pregnancy / number of FET cycles*100%. Early miscarriage rate = number of cycles with early miscarriage / number of cycles with clinical pregnancy *100%. Live birth rate = number of cycles with live births / number of transfer cycles*100%.

### Statistical analysis

Statistical analysis was performed using the Empower Stats software base on R language. Continuous variables were presented as mean ± SD, and categorical variables were presented as N (%). Comparisons of these variables between groups were performed using a one-way ANOVA and χ2 tests for categorical variables. Smooth curve fitting was performed to assess if there was any non-linear relationship between endometrial thickness and pregnancy outcomes. Then a segmented regression model was used to analyze the threshold effect between endometrial thickness and pregnancy outcomes. P < 0.05 was considered statistically significant.

## Results

A total of 10,165 HRT-FET cycles were analyzed and reported here with the overall implantation rate of 46.45%, clinical pregnancy rate of 62.11%, and live birth rate of 51.08%.

All patients were grouped according to their endometrial thickness on the ET day. Patient demographics and characteristics are shown in Table 1. There were significant differences between the age, infertility duration, BMI and percentage of primary infertility of the women in each group, but there was no difference in the number and type of embryos transferred. (Table 1)

**Table 1:** Characteristics and periodic features in patients grouped by endometrial thickness on the embryo transfer day.

Group	EN ≤6 mm	EN 6–7 mm	EN 7–8 mm	EN 8–9 mm	EN 9–10 mm	EN 10–11 mm	EN 11–12 mm	EN 12–13 mm	EN>13 mm	P-value
N	235	445	1526	3338	2299	1218	615	291	198	
Age	34.06 ± 6.19	33.54 ± 6.09	32.36 ± 5.92	31.17 ± 5.60	30.99 ± 5.42	31.08 ± 5.27	31.69 ± 5.71	31.84 ± 5.49	32.67 ± 6.50	<0.001
Duration of infertility	3.44 ± 2.82	3.73 ± 3.13	4.06 ± 2.99	4.05 ± 2.89	4.30 ± 3.30	4.31 ± 3.27	4.62 ± 3.44	4.38 ± 3.53	5.11 ± 3.57	<0.001
BMI	22.74 ± 3.07	23.18 ± 3.74	23.02 ± 3.42	22.87 ± 4.24	23.04 ± 4.63	23.33 ± 7.47	23.47 ± 3.32	23.69 ± 3.70	23.80 ± 3.64	<0.001
No. of embryo transferred	1.81 ± 0.43	1.82 ± 0.43	1.83 ± 0.41	1.85 ± 0.38	1.85 ± 0.38	1.84 ± 0.37	1.86 ± 0.35	1.85 ± 0.37	1.81 ± 0.43	0.273
Infertility type										<0.001
Primary Infertility	25.33% (58/229)	26.09% (114/437)	41.26% (621/1505)	52.78% (1727/3272)	56.01% (1267/2262)	59.00% (705/1195)	60.23% (362/601)	57.19% (163/285)	56.48% (109/193)	
Secondary infertility	74.67% (171/229)	73.91% (323/437)	58.74% (884/1505)	47.22% (1545/3272)	43.99% (995/2262)	41.00% (490/1195)	39.77% (239/601)	42.81% (122/285)	43.52% (84/193)	
Embryo type										0.433
Embryos in cleavage stage	76.17% (179/235)	74.83% (333/445)	72.94% (1113/1526)	72.44% (2418/3338)	71.58% (1645/2298)	70.11% (854/1218)	73.98% (455/615)	71.82% (209/291)	73.23% (145/198)	
Blastocyst	23.83% (56/235)	25.17% (112 /445)	27.06% (413/1526)	27.56% (920/3338)	28.42% (653/2298)	29.89% (854/1218)	26.02% (160/615)	28.18% (82/291)	26.77% (53/198)	

### Comparison of clinical outcomes between different groups

The implantation rate, the early miscarriage rate, clinical pregnancy rate and live birth rate were significantly different among groups that had different endometrial thickness (P < 0.001). In addition, there were no statistical significances among in the ectopic pregnancy rate, delivery gestational weeks and newborn birth weight between groups (p > 0.05) (Table 2).

**Table 2:** Clinical outcomes in patients grouped by endometrial thickness on the embryo transfer day.

Group	EN ≤ 6 mm	EN 6–7 mm	EN 7–8 mm	EN 8–9 mm	EN 9–10 mm	EN 10–11 mm	EN 11–12 mm	EN 12–13 mm	EN >13 mm	P-value
N	235	445	1526	3338	2299	1218	615	291	198	
Implantation rate	27.76% (118/425)	28.94% (235/812)	39.26% (1097/2794)	49.50% (3059/6180)	48.81% (2078/4257)	51.09% (1098/2245)	51.31% (587/1144)	50.65% (273/539)	46.37% (166/358)	<0.001
Clinical pregnancy rate	38.46% (90/234)	44.14% (196/480)	54.24% (825/1521)	65.83% (2192/3330)	65.22% (1498/2297)	64.86% (788/1215)	67.48% (415/615)	67.01% (195/291)	62.63% (124/198)	<0.001
Ectopic pregnancy rate	4.44% (4/90)	2.55% (5/196)	1.82% (15/825)	1.32% (29/2192)	1.67% (25/1498)	1.52% (12/788)	0.72% (3/415)	1.54% (3/195)	1.61% (2/124)	0.313
Early miscarriage rate	25.56% (23/90)	17.86% (35/196)	13.58% (112/825)	10.40% (228/2192)	10.35% (155/1498)	9.90% (78/788)	10.12% (42/415)	12.82% (25/195)	16.94% (21/124)	<0.001
Live birth rate	25.11% (59/235)	32.13% (143/445)	42.60% (650/1526)	54.70% (1826/3338)	54.76% (1259/2299)	54.35% (662/1218)	55.77% (343/615)	53.26% (155/291)	47.98% (95/198)	<0.001
Gestational week at delivery	37.34 ± 2.35	37.67 ± 2.36	37.44 ± 2.64	37.57 ± 2.50	37.61 ± 2.43	37.54 ± 2.49	37.49 ± 2.49	37.60 ± 2.35	37.66 ± 1.63	0.912
Newborn birth weight	3114.66 ± 684.58	3196.92 ± 655.47	3099.89 ± 731.15	3106.46 ± 691.47	3119.72 ± 678.25	3153.67 ± 698.83	3084.11 ± 674.71	3137.08 ± 658.12	3224.74 ± 668.59	0.456

### Multivariable logistic regression used to evaluate the association between endometrial thickness and pregnancy outcomes

In order to control the impact of age, the duration of infertility, BMI and the infertility type (primary or secondary) on pregnancy outcomes, we performed multivariable logistic regression analysis to evaluate the association between endometrial thickness and pregnancy outcomes. After making adjustments based on the above factors, significant associations were found between endometrial thickness and implantation rate(adjusted odds ratio [aOR]: 1.08; 95% confidence interval [CI]: 1.06–1.10, p < 0.0001), clinical pregnancy rate(aOR: 1.10; 95% CI: 1.07–1.14, p < 0.0001) and live birth rate (aOR: 1.09; 95% CI: 1.06–1.12, p < 0.0001). The associations between endometrial thickness and early miscarriage rate, ectopic pregnancy rate, gestational weeks at delivery and newborn birth weight were not significant (Table 3).

**Table 3:** Associations between endometrial thickness and clinical outcomes of HRT-FET cycles using multivariable logistic regression analysis. All adjusted for age, the duration of infertility, body mass index, infertility type, and the number type of embryos transferred.

Item	Non-adjusted			Adjusted I			Adjusted II		
	OR	95% CI	P	OR	95% CI	P	OR	95% CI	P
Implantation rate	1.10	(1.08, 1.12)	< 0.0001	1.08	(1.06, 1.10)	<0.0001	1.08	(1.06, 1.10)	< 0.001
Clinical pregnancy rate	1.13	(1.10, 1.15)	< 0.0001	1.10	(1.07, 1.14)	<0.0001	1.10	(1.07, 1.14)	< 0.001
Ectopic pregnancy rate	0.90	(0.79, 1.02)	0.1075	0.92	(0.80, 1.06)	0.2369	0.92	(0.80, 1.06)	0.2427
Early miscarriage rate	0.95	(0.90, 0.99)	0.0284	0.96	(0.91, 1.01)	0.1101	0.96	(0.91, 1.01)	0.1307
Live birth rate	1.12	(1.09, 1.14)	<0.0001	1.09	(1.07, 1.12)	<0.0001	1.09	(1.06, 1.12)	<0.001
Gestational age at delivery	0.0	(-0.0, 0.1)	0.5420	0.0	(-0.0, 0.1)	0.2283	0.0	(-0.0, 0.1)	0.2252
Newborn birth weight	7.2	(-4.9, 19.3)	0.2426	10.3	(-2.1, 22.7)	0.1027	10.2	(-2.2, 22.6)	0.1055

### Curve fitting

**Fig 1:**
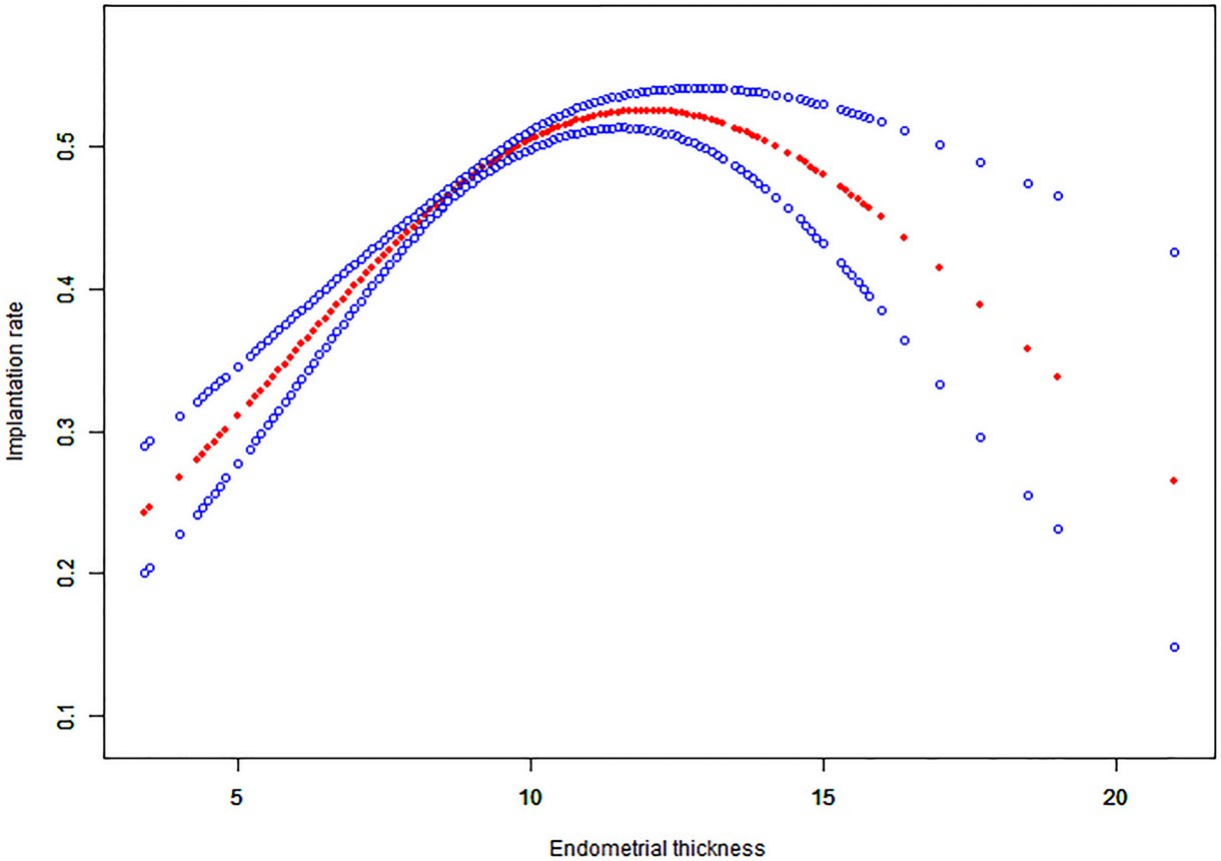
Associations between endometrial thickness and implantation rate of HRT-FET cycles. The threshold was identified, and a nonlinear association between endometrial thickness and the implantation rate was found (P<0.001) using a generalized additive model (GAM). The solid-dotted red line represents the smooth curve that fits between variables. Blue curves represent the upper and lower limits of the 95% CI. All were adjusted for age, the duration of infertility, body mass index, infertility type, and the number and type of embryos transferred.

**Fig 2:**
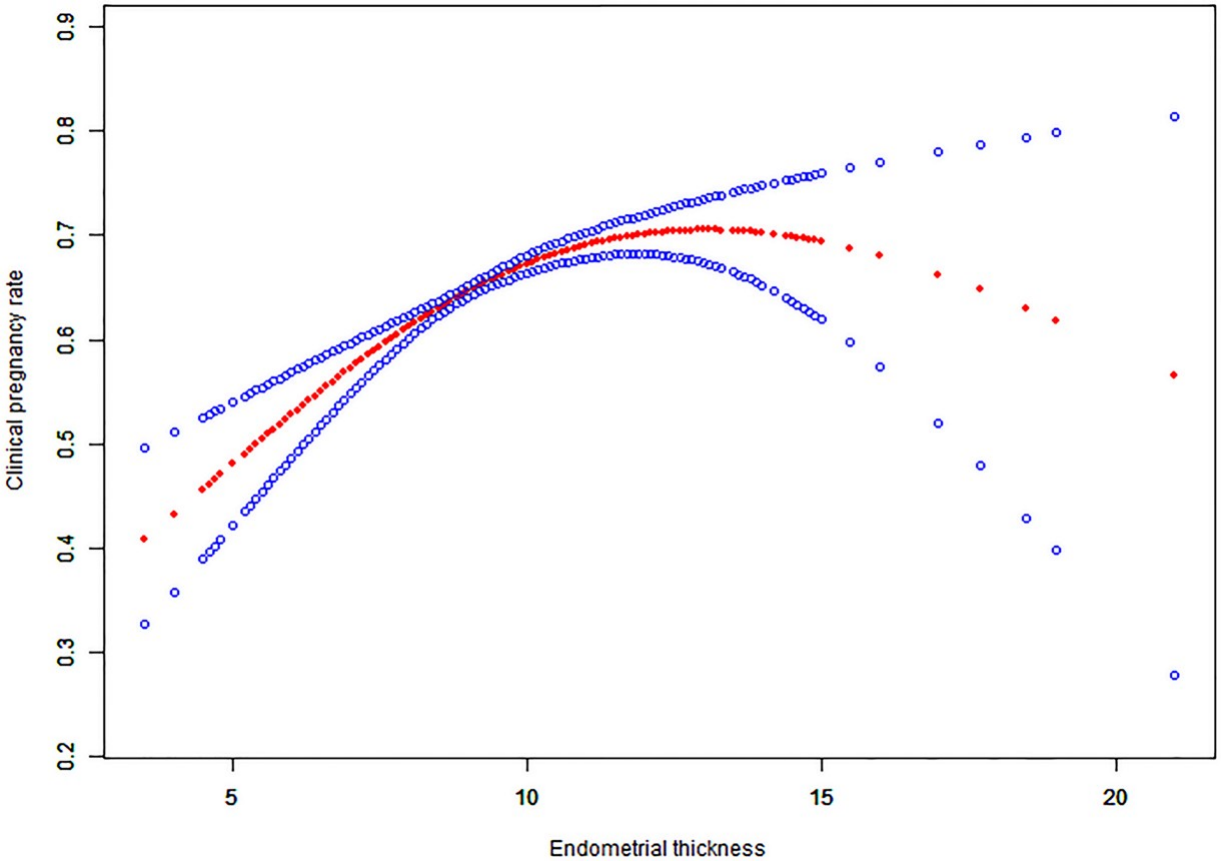
Associations between endometrial thickness and clinical pregnancy rate of HRT-FET cycles. The threshold was identified, and a nonlinear association between endometrial thickness and the clinical pregnancy rate was found (P<0.001) using a GAM. The solid-dotted red line represents the smooth curve that fits between variables. Blue curves represent the upper and lower limits of the 95% CI. All were adjusted for age, the duration of infertility, body mass index, infertility type, and the number and type of embryos transferred.

**Fig 3:**
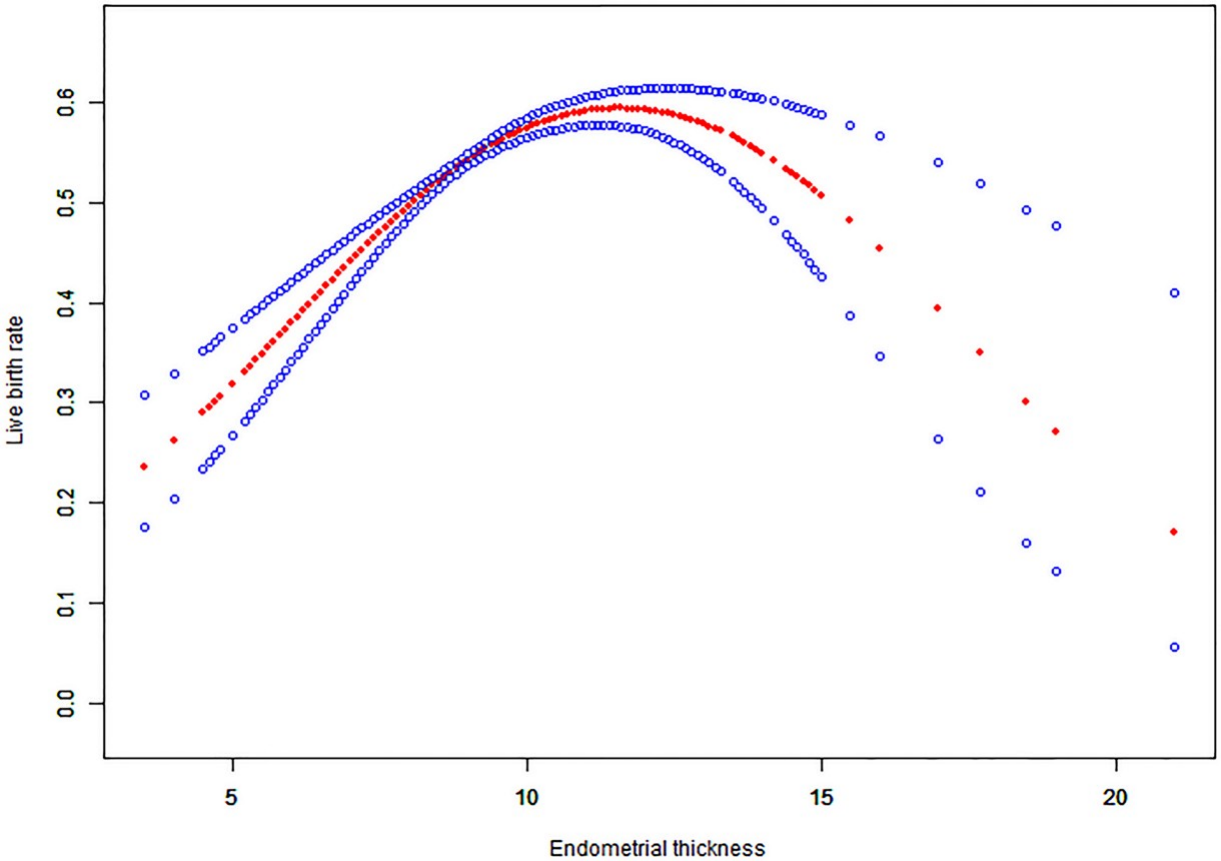
Associations between endometrial thickness and live birth rate of HRT-FET cycles. The threshold was identified, and a nonlinear association between endometrial thickness and the live birth rate was found (P<0.001) using a GAM. The solid-dotted red line represents the smooth curve that fits between variables. Blue curves represent the upper and lower limits of the 95% CI. All were adjusted for age, the duration of infertility, body mass index, infertility type, and the number and type of embryos transferred.

For continuous variables such as endometrial thickness, the implantation rate, clinical pregnancy rate and live birth rate, the fitted curves are presented in Figs 1–3, respectively. With the increase of endometrial thickness, the implantation rate, clinical pregnancy rate and live birth rate initially went up and then down (Figs 1–3).

### Threshold effect analysis results

The threshold effect analysis of endometrial thickness and the implantation rate, clinical pregnancy rate and live birth rate are presented in Table 4. The endometrial thickness is a non-linear significant predictor of clinical outcomes, and its turning point is 8.7 mm. The implantation rate, clinical pregnancy rate and live birth rate increased by 32%, 36% and 45%, respectively with the increase of each millimeter increment of endometrial thickness up to 8.7 mm. When the endometrial thickness was ≥ 8.7 mm, the clinical outcomes did not increase significantly with but rather tended to be stable.

**Table 4:** Threshold effect analysis of endometrial thickness and clinical outcomes of HRT-FET cycles using piece-wise linear regression method.

Clinical outcomes	Turning point of endometrial thickness	Effect size (OR)	95% CI	P value
Clinical pregnancy rate	<8.7	1.36	(1.26, 1.47)	< 0.0001
	≥8.7	1.02	(0.99, 1.06)	0.2051
Live birth rate	<8.7	1.45	(1.33, 1.57)	< 0.0001
	≥8.7	1.00	(0.97, 1.04)	0.9792
Implantation rate	<8.7	1.32	(1.24, 1.40)	< 0.0001
	≥8.7	1.01	(0.99, 1.04)	0.2977

### Influence of the delta between the endometrial thickness on the transfer day compared with the thickness at the starting of progesterone day on the clinical outcome

Between the endometrial thickness on the transfer day compared with the thickness at the starting of progesterone day, endometrial thickness had no changed cycles, the increased and compaction cycles accounted for 18.88%, 38.05%, 47.80% of the total number of cycles, respectively. The clinical pregnancy rate of cycles with compaction endometrial thickness is lower than the other two groups (P<0.001) (Table 5).

**Table 5:** Influence of the delta between the endometrial thickness on the transfer day compared with the thickness at the starting of progesterone day on the clinical outcome.

Delta of endometrial thickness	Statistics	Clinical pregnancy rate
No change	18.88% (1919/10165)	60.19% (1155/1919)
Increase	38.05% (3868/10165)	66.55% (2553/3868)
Compaction	47.80% (4859/10165)	53.82% (2615/4859)
*P*		<0.001

## Discussion

Endometrial receptivity is the key factor affecting the pregnancy outcomes of embryo transfer cycles [9]. It has the advantage of: being non-invasive, simplicity, convenience, cost-effectiveness, repeatability as well as other advantages. Using transvaginal ultrasonography to measure endometrial thickness is often used to help assess the timing of endometrial transformation and the endometrial receptivity [9, 10]. However, there is no consensus on the relationship between endometrial thickness and pregnancy outcomes. The endometrial thickness was measured at different time points during previous studies, such as on the day of hCG administration, on the day of oocyte retrieval or on the day embryos transferred [11–14]. In addition to this, the published studies also varied in other factors, for example, controlled ovarian stimulation protocols, FET protocols, number of embryos transferred, and the type of embryo transferred (cleavage stage embryo or blastocyst) [14–17]. Many factors could affect endometrial receptivity in fresh IVF cycles and natural cycle FETs, such as excessive estrogen levels, elevated endogenous progesterone, and LH surge. Since such factors can be confounding and affect the reliability of the study results, the relationship between endometrial thickness and IVF outcomes has been a subject of much debate for several decades. Recently, a meta-analysis including 22 studies concluded that there seems to be no justification for using to use endometrial thickness as a tool to help people decide on cycle cancellation, freeze-all or refraining from further IVF treatment [18]. Another meta-analysis that included 4,922 cycles from 14 studies was not able to draw a convincing conclusion on the relationship between endometrial thickness and the pregnancy rate in IVF [19].

As far as we know, previous studies only focused on whether endometrial thickness affects clinical outcomes or not. The study of K.E.Liu et al. calculated the ORs and their 95% CIs for ongoing pregnancy outcomes with different cut-off values of EMT, and the authors concluded that ongoing pregnancy likelihood was significantly higher in patients with EMT ≥ 9 mm as compared to those who had EMT 3–8 mm [14]. A retrospective study of FET cycles by Tarek El-Toukhy et al. compared patients whose endometrial thickness were < 7 mm, 7–8 mm, 9–14 mm and > 14 mm, and found that those with the endometrial thickness of 9–14 mm on the day of progesterone supplementation had higher rates of implantation and pregnancy rates [5]. As with most of the previous studies, in the study of Zhiqin Bu et al., all patients were classified into three groups (Group A, EMT ≤ 8 mm; Group B, EMT 9–13 mm; Group C, EMT ≥ 14 mm) according to the endometrial thickness on the ET day. Patients who had thin endometrial thickness in Group A had significantly lower rates in clinical pregnancy and live birth than those in Group B or C [9]. In our study, we found significant associations between endometrial thickness and the rates of implantation (aOR: 1.08; 95% CI: 1.06–1.10, p < 0.0001), clinical pregnancy (aOR: 1.10; 95% CI: 1.07–1.14, p < 0.0001) and live birth (aOR: 1.09;95% CI: 1.06–1.12, p < 0.0001). The curve fitting analysis further revealed a quantitative relationship between endometrial thickness and clinical outcomes. The cut-off value of the endometrial thickness was 8.7 mm. With every millimeter increment of endometrial thickness up to 8.7 mm, the implantation rate, clinical pregnancy rate and live birth rate increased by 32%, 36% and 45%, respectively. However, the clinical outcomes tended to remain stable without further increasing when the endometrial thickness is ≥ 8.7 mm.

It is noteworthy that this study is the first to report a minimum threshold of endometrial thickness for optimal pregnancy outcomes. Several studies in the past merely reported a relatively broad range of endometrial thicknesses that were considered optimal for pregnancy outcomes while the classification of endometrial thickness in their studies was arbitrary, being mainly based on clinical experience or references [5, 9, 14], while the range of endometrial thickness in each group was also very large, thus making these studies unlikely of being able to provide good guidance for clinical practice. The result of threshold effect analysis showed that 8.7 mm was the lowest threshold of endometrial thickness for the optimal live birth rate of 50.61%. When the endometrial thickness was larger than 8.7 mm, the increase of the live birth rate was subtle and tended to be stable. The live birth rate at this point was considered optimal, and thus the range of endometrial thickness relating to the optimal live birth rate could therefore be obtained. Combining the threshold effect analysis results and the curve-fitting pattern, our data showed that the live birth rate would be optimal when the endometrial thickness was within the range of 8.7–14.5 mm. In the group whose endometrial thickness had reached beyond 14.5 mm, the implantation rate, clinical pregnancy rate and live birth rate were slightly lower, a fact which was consistent with the curve fitting results. If the endometrium was too thin or too thick, the live birth rate would be reduced. This is the first study to explore what is the best range of endometrial thickness for optimal live birth rate from the statistical perspective, and the results of it have great clinical significance.

Although the rates of clinical pregnancy and live birth were lower when the endometrial thickness was 7–8 mm, the outcomes in this group were still reasonably acceptable. The rates of clinical pregnancy and live birth significantly decreased to 44.14% and 32.13%, respectively, when the endometrial thickness was 6–7 mm, and then to 38.46% and 25.11%, respectively, when the endometrial thickness was ≤ 6 mm. These results may serve as a guide for clinicians and patients when facing a persistently thin endometrium. In the two groups which had an endometrial thickness of ≤ 7 mm, the proportion of patients with primary infertility was significantly lower than in the group with endometrial thickness of > 7 mm, suggesting that the thin endometrium might be the result of a prior uterine operation that caused secondary infertility.

In our study, we did not find any significant effect of endometrial thickness on pre-term delivery or neonatal birth weight. In the study of Ribeiro V C et al, the gestational age seemed unaffected by endometrial thickness, a point which is consistent with our study as well. However, they found birth weight z-scores varied significantly depending on the different endometrial thickness [13]. A possible explanation for this might be that they investigated the effect of endometrial thickness on pregnancy outcomes in the fresh cycles, and the timing of the endometrial thickness measurement was not clearly reported.

In addition, we noted that some recent studies [20, 21] that have looked at the effect of endometrial changes between the transfer day and the starting of progesterone day, on clinical pregnancy outcomes. Our results suggest that endometrial compaction on transplantation day has a negative effect on clinical pregnancy outcomes. At present, there is no clear evidence that the increase or decrease of the endometrial thickness between the transfer day and the starting of progesterone day on the clinical outcome, which is worthy of further study.

To the best of our knowledge, this is the largest study to date that evaluates the effects of endometrial thickness on the outcomes of patients participating in HRT-FET cycles. In addition, as a result of having a large sample size, more consistent embryo grading, consistent endometrial thickness measurement and ultrasound instruments, the impact of these factors on the study results was minimized. We also studied the relationship between endometrial thickness and clinical outcomes in a more homogenous study population that underwent the same HRT protocol. The endometrial implantation window was more uniform, only relating to the timing of progesterone administration that was fixed in this study. According to common knowledge, embryo quality and endometrial preparation protocol are the two most critical factors which affect the pregnancy outcomes of FET. In all the cycles in this study, at least one good-quality embryo was transferred, which excluded the effect of embryo quality on pregnancy outcome. Since no minimal cut-off value of endometrium thickness was defined in the original study protocol, the effect of the whole spectrum of endometrial thicknesses in HRT-FET could be studied. Nodal segmentation of endometrial thickness was also not performed. Moreover, the analysis was adjusted for age, the duration of infertility, body mass index, and type of infertility and number (cleavage stage embryos or blastocysts) of embryos transferred using multivariable logistic regression, all of which contributed to the robust results of this study.

At the same time, this study still had its limitations. As with other relevant clinical studies, an important limitation of this study was the retrospective design, even though we established strict inclusion and exclusion criteria and adjusted confounding factors to control bias through multivariable logistic regression. In addition, the ultrasound monitoring of endometrial thickness might have some measurement imprecision that occur regardless of how experienced the ultrasonographers may be. There were only 37 patients with an endometrial thickness above the maximum threshold. Which suggests that more HRT-FET cycles especially those with thick endometrium are to be collected in future studies, with the hope of being able to determine an optimal range of endometrial thickness for pregnancy outcomes. In the future, we will continue analyze the data of our center to evaluate the difference between endometrial thickness on the first progesterone day and that on the ET day, and its correlation with the pregnancy outcomes, so as to provide guidance for clinical practice.

In conclusion, in the HRT-FET cycles, a satisfactory live birth rate can be obtained when endometrial thickness is kept within the range of 8.7–14.5 mm. If the endometrial thickness is too thin or too thick, the live birth rate will be reduced. Based on these research results, It is recommended to medical practitioners that transferring embryos should be conducted when the endometrial thickness reaches 8.7 mm, and there is no need to wait for the endometrial to get any thicker. The risk of preterm labor and neonatal birth weight were not significantly correlated with endometrial thickness.

## Supporting information

S1 File (DOCX)

S2 File (DOCX)

S1 Data (XLSX)
