# Supplementary material for: The effect of endometrial thickness on pregnancy outcomes of frozen-thawed embryo transfer cycles which underwent hormone replacement therapy
Source: PLoS One. 2020 Sep 24;15(9):e0239120. doi: 10.1371/journal.pone.0239120 (PMC7513995; doi:10.1371/journal.pone.0239120)
Supplement: S1 File — (DOCX) [file pone.0239120.s001.docx]

**郑州大学生命科学伦理审查委员会**

伦 理 审 查 报 告

| **项目名称：**子宫内膜厚度对激素替代治疗冷冻胚胎移植周期妊娠结局的影响  **项目负责人：**张少娣  **伦理审查意见：**    经郑州大学生命科学伦理审查委员会审查，该项目（伦理编号：ZDRMYY-LL-2018051326）研究内容和过程遵循国际及国家颁布的有关生物医学研究的伦理要求，同意该项目开展。  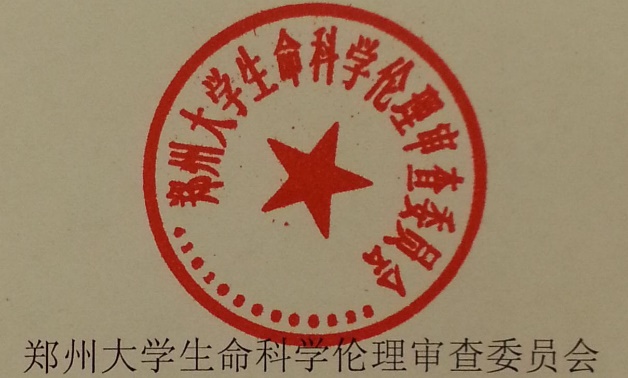  2018年 5月16 日 |
| --- |
