# Supplementary material for: The effect of endometrial thickness on pregnancy outcomes of frozen-thawed embryo transfer cycles which underwent hormone replacement therapy
Source: PLoS One. 2020 Sep 24;15(9):e0239120. doi: 10.1371/journal.pone.0239120 (PMC7513995; doi:10.1371/journal.pone.0239120)
Supplement: S2 File — (DOCX) [file pone.0239120.s002.docx]

Life science ethics review committee of Zhengzhou university

Report of the audit

| Project Name：Comparison between two forms of vaginally administered progesterone combined with oral dydrogesterone for luteal phase support in hormone replacement frozen-thawed embryo transfer (HRT-FET) cycles- A real world study |
| --- |
| Project Leader：Zhangshaodi |
| Opinion on ethical review:  After the review of the life science ethics review committee of Zhengzhou university, the research (Ethical code: ZDRMYY-LL-2019081428) content and process of this project follow the international and national ethical requirements on biomedical research and agree to carry out this project.    Life science ethics review committee of Zhengzhou university  May 30, 2019 |
